# Machine learning-based land-use regression models for predicting carbon dioxide concentrations in San Francisco Bay area

**DOI:** 10.1007/s12665-025-12582-w

**Published:** 2025-09-26

**Authors:** Anna C. Smith, Linfeng Li, Jiansheng Xiang, Fangxin Fang

**Affiliations:** Department of Earth Science and Engineering, Imperial College London, SW7 2AZ, London, United Kingdom

**Keywords:** Land use regression, Carbon dioxide, XGBoost, CNN, Urban

## Abstract

Carbon dioxide (CO_2_) is a key driver of anthropogenic climate change and cities have been identified as major sources of emissions. Urbanization and land use change are associated with rising urban CO_2_ emissions, highlighting the need to study spatiotemporal trends in intraurban CO_2_ to inform sustainable city planning. This study investigates the use of land use regression (LUR) to predict intraurban CO_2_ concentrations, using data from the BEACO_2_N monitoring network in the San Francisco Bay Area. Additionally, LUR is compared to machine learning (ML) algorithms capable of capturing non-linear relationships, representing a two-fold novel contribution. Model performance is evaluated using reserved data from training sensors as well as unseen sensor locations. For training sensors, extreme gradient boosting (XGBoost) and a convolutional neural network (CNN) achieved the highest predictive accuracy (R²=0.58), outperforming traditional LUR (R²=0.34). XGBoost and CNN also outperformed traditional LUR for unseen sensor locations, accounting for up to 42% of the variability in observed CO_2_ concentrations. These models offer insight into urban land use and carbon dynamics, supporting more informed approaches to urban planning and decarbonization.

**Supplementary Information:**

The online version contains supplementary material available at 10.1007/s12665-025-12582-w.

## Introduction

Urbanization is a key trend in the twenty-first century, and 70% of energy-related CO_2_ emissions globally are associated with urban areas (Intergovernmental Panel on Climate ClimateChange (IPCC) 2023). Currently, over half of the world’s population lives in cities, and by 2100 this rate is projected to increase up to 80–90% (Riahi et al. 2017). Carbon dioxide (CO_2_) is a critical greenhouse gas (GHG) produced during the combustion of fossil fuels and a key driver of anthropogenic climate change. Research has identified positive correlations between urbanization and CO_2_ emissions (Li and Yan 2024; Poumanyvong and Kaneko 2010; Wang 2018). Long-term analysis of urban expansion in rapidly growing cities has shown that land use change can lead to both increased emissions and reduced carbon storage, illustrating how urbanization can simultaneously intensify sources of CO₂ and diminish natural sinks (Li and Yan 2024). Therefore, cities must be recognized as key contributors to climate change, highlighting the need to mitigate their climate impact. Better understanding trends and variability in intraurban CO_2_ is important to inform decision-makers in the development of urban decarbonization strategies (Mitchell et al. 2018; Wang 2018; Qian et al. 2022; Hu et al. 2023).

Intraurban CO_2_ demonstrates considerable spatiotemporal variability. The heterogeneous nature of urban development and land use results in spatial variability of ambient CO₂ levels across cities (Wang 2018). Urban areas are known to be net sources of CO_2_, exhibiting distinct seasonal and diurnal patterns: CO_2_ levels peak in the mornings and are lowest in the evenings, with less pronounced fluctuations in the summer compared to the winter (Coutts et al. 2007; Velasco et al. 2005). An increase in wintertime emissions can be linked to more heating fuel combustion and less vegetation cover (Bergeron and Strachan 2011; Coutts et al. 2007). Patterns in ambient CO_2_ are also closely related to traffic volumes, with an increase in emissions observed during rush hour periods (Coutts et al. 2007). Additionally, suburban areas generally experience lower ambient CO_2_ concentrations than urban centres (Bergeron and Strachan 2011; Velasco and Roth 2010). Mitchell et al. (2018) identified an increase in emissions resulting from suburban development and population growth in rural areas. Li and Stouffs (2024) studied the impact of land use and land cover (LULC) changes on urban carbon storage, highlighting the importance of urban planning and carbon modelling on decarbonisation.

Unlike air pollutants measured by air quality sensors, there is a lack of widespread sensors to measure ambient CO_2_. Instead, CO_2_ emissions are typically calculated using aggregated emissions or energy consumption data to assign responsibility to governmental or corporate entities in a bottom-up way (Duren and Miller 2012; Mitchell et al. 2018). Emissions data indeed can be collected in a top-down fashion which requires global or local concentration measurement (National Academies of Sciences, 2022). Efforts to establish CO_2_ monitoring networks are rising, with recent research demonstrating urban CO_2_ sensor deployment primarily in the U.S. (Bréon et al. 2015; Briber et al. 2013; Duren and Miller 2012; Lauvaux et al. 2016; Mitchell et al. 2018; Rice and Bostrom 2011). A key example is the Megacities Carbon Project, which represents a significant step toward long-term, multisite CO_2_ monitoring in megacities worldwide (Duren and Miller 2012). Despite this progress, many existing networks are limited in sensor coverage, with some featuring only one to five sensors (Bréon et al. 2015; Briber et al. 2013; Helfter et al. 2016; Mitchell et al. 2018; Rice and Bostrom 2011). Additionally, most networks have been established recently, offering a limited historical record of ambient CO_2_ concentrations (Duren and Miller 2012). This leaves gaps in understanding urban carbon dynamics and underscores the need for more long-term, spatially distributed CO_2_ monitoring networks (Mitchell et al. 2018).

Spatiotemporally distributed measurements are key to modelling intraurban ambient gas concentrations using modelling techniques such as land use regression (LUR). LUR is a specialized application of multiple linear regression used to estimate ambient air pollution. It operates under the principle that environmental features, such as land use, population density, road networks, topography, and meteorological conditions are relevant predictors for air pollutant concentrations (Li et al. 2021). The most common use of LUR is to produce exposure assessments for epidemiological studies that predict what levels of air pollution survey participants may be exposed to at unmonitored locations, such as their places of residence (Larkin et al. 2023; Li et al. 2021; Ryan and LeMasters 2007). Researchers have used LUR to predict concentrations of nitrogen dioxide (NO_2_), ambient respirable suspended particulates (PM10), fine suspended particulates (PM2.5), ozone (O_3_) and carbon monoxide (CO) (Larkin et al. 2023; Li et al. 2021; Wong et al. 2021).

In a literature review synthesizing the development of LUR models, Ryan and LeMasters (2007) analysed 12 studies and found that independent variables used in LUR can be broadly categorized into four groups: (1) road type, (2) traffic count, (3) elevation, and (4) land cover; traffic count was generally the most important predictor variable. LUR can achieve considerable accuracy, and Ryan and LeMasters (2007) found that the LUR models reviewed in the study accounted for between 54% and 81% of the variability in air pollutant concentrations. Furthermore, the integration of machine learning (ML) and deep learning (DL) algorithms, particularly extreme gradient boosting (XGBoost), has proven capable of improving upon LUR accuracy (Wong et al. 2021). This improvement is due to the fact that tree-based models, like XGBoost, can account for non-linear relationships among the predictors and target variable.

In addition to tree-based models such as XGBoost, it is possible to incorporate convolutional layers into machine learning models to better capture spatial variation. The prediction task presented in this study is based on sparse measurements on an unstructured grid of nodes, which limits the applicability of more advanced convolutional neural networks like those used by Li et al. (2025), where ResNet and U-Net architectures were employed to simulate land surface temperature on a regular, image-like grid in urban environments. However, as demonstrated by Cheng et al. (2020), for fluid problems defined on unstructured grid, a one-dimensional representation of the unstructured-grid nodes can enable 1D convolutional layers to learn spatial features.

One major advantage of LUR is its ability to capture fine-scale spatial and temporal patterns (Larkin et al. 2023; Ryan and LeMasters 2007). Intraurban air pollution is characterized by high spatial and temporal variability due to seasonal and daily variations in traffic and meteorological conditions, as well as the decay of pollutants over space and time (Larkin et al. 2023; Ryan and LeMasters 2007). Therefore, granular data is key when attempting to accurately capture spatiotemporal variability in intraurban air pollution. Limitations of LUR include poor transferability between cities and limited global generalizability, partly due to uneven spatial distributions of sensors (Larkin et al. 2023; Li et al. 2021). The performance of the models is sensitive to the quality and quantity of training data, the location of sensors, and the choice of predictor variables (Ryan and LeMasters 2007).

A review of existing literature has produced no evidence of using LUR to model intraurban CO_2_ concentrations, nor any discussion about the feasibility of such an approach. Possible explanations include that CO_2_ is a GHG rather than an air pollutant associated with human health risks. Therefore, it has little relevance to LUR’s most common application of creating exposure assessments for epidemiological studies. Furthermore, the scarcity of long-term, spatially distributed urban CO_2_ monitoring networks may have stalled the development of LUR models that rely on temporally and spatially distributed CO_2_ data. This study aims to address this research gap by developing an LUR model to predict intraurban CO_2_.

To understand how land use affects intraurban CO_2_, this study develops a linear LUR model and two non-linear ML models to predict intraurban CO_2_ concentration. The viability and performance of both the linear and the non-linear models are tested and discussed as a case study in the San Francisco Bay Area. The application of LUR model to ambient CO_2_ concentration prediction is examined for the first time. Next, the performance of ML-based models in explaining non-linear features is explored. Moreover, the site-to-site transferability of the proposed models is tested using data from unseen locations. Finally, the city layout impact on CO_2_ concentration is analysed and the implication of the model outcomes on urban planning is discussed.

## Methodology

### Study site: BEACO_2_N CO_2_ network

**Fig. 1:**
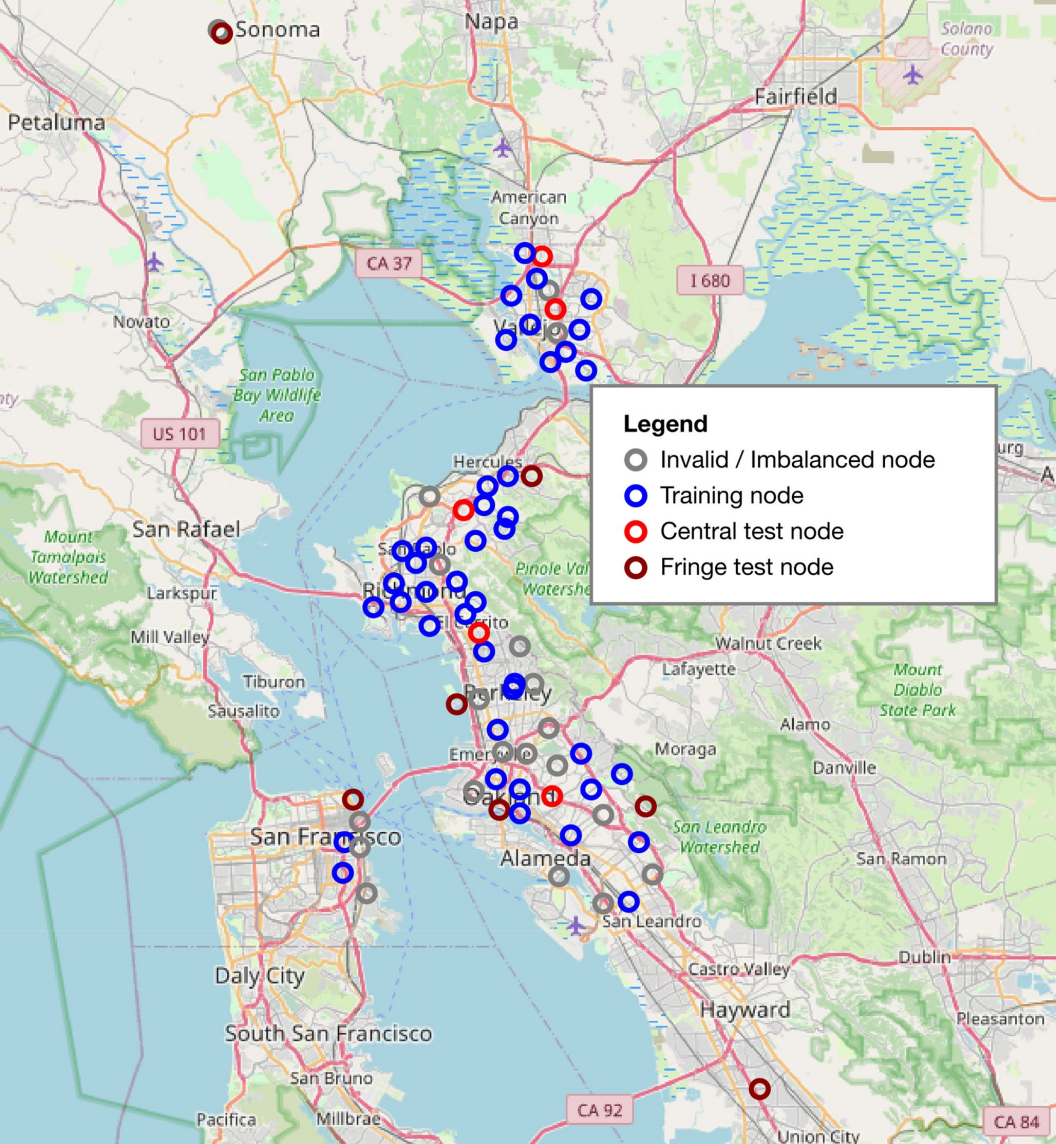
BEACO_2_N sensor locations and assigned groups

The Berkeley Environmental Air-quality & CO_2_ Network (BEACO_2_N) was identified as a promising source of spatiotemporally distributed intraurban CO_2_ data. The sensor network was established in the San Francisco Bay Area by a team of researchers at the University of California, Berkeley (Shusterman et al. 2016). A total of 74 unique sensors, or “nodes”, collected real-time CO_2_ data across the San Francisco Peninsula, the East Bay, and North Bay between 2012 and 2024, as shown in Fig. 1. These sensors also recorded data for NO, NO_2_, O_3_, CO, aerosols, and meteorological conditions such as temperature, pressure, and relative humidity. The data has a temporal resolution of one minute and a spatial resolution of approximately one mile, which was the finest spatial and temporal resolution identified among any intraurban CO_2_ monitoring network. The downloaded BEACO_2_N dataset comprised over 2.4 million raw observations. CO_2_ and meteorological data were temporally aggregated for modelling by calculating daily averages, provided a sensor recorded at least 18 out of 24 h of valid data in a single day.

The number of active reporting sensors varied throughout the 12-year period. Sensors were added and removed over time, increasing from 12 in 2012 to a peak of 54 in 2023. Some sensors also experienced periods of malfunction or inactivity, leading to additional temporal inconsistencies in the data. Invalid observations for CO_2_ and meteorological variables were discarded, as were data from the COVID-19 years of 2020 and 2021. After filtering out invalid data and excluding daily averages with less than 18 h of valid observations, the dataset yielded 67,044 observations from 65 distinct nodes.

### Predictor variables

The choice of candidate predictor variables was informed by previous LUR studies by Larkin et al. (2023), Lee et al. (2017), and Li et al. (2021). Variables related to land use, road traffic, vegetation, urbanization, and meteorology were chosen and downloaded based on relevance and availability. In addition, data for industrial areas was supplemented, given that these sites and activities are generally associated with elevated carbon emissions. Table 1 summarizes the features and data sources used.

**Table 1:** A list of feature variables to be selected in modelling

Feature Category	Potential Variable	Year	Source
*buffer radii 50 m*,* 100 m*,* 200 m*,* 300 m*,* 500 m*,* 1000 m*,* 1500 m*,* 2000 m*,* 3000 m*,* 4000 m*,* 5000 m*			
Land Use *total area [m* ^*2*^ *] in buffer*	Built Area Rangeland Trees Water Bare Ground Crops Flooded Vegetation	2021	*ESRI Sentinel-2 10 m Land Use/Land Cover*
Industrial Areas *total area [m* ^*2*^ *]in buffer*	Industrial	2021–2023	*California General Plan Land Use*
Annual Average Daily Traffic (AADT) *total count [AADT] in buffer*	AADT	2022	*Caltrans 2022 Traffic Volumes (AADT)*
Roads *total length [m] in buffer*	Road Length	2022	*U.S. Census Bureau 2022 California Roads TIGER/Line*
Normalized Difference Vegetation Index (NDVI) *mean NDVI in buffer*	NDVI	2022	*Landsat 8–9 OLI/TIRS C2 L2 Surface Reflectance-derived NDVI*
Population Density *people per km*^*2*^ *in buffer*	Population Density	2022	*California Hard-to-Count Index* *U.S. Census Bureau 2022 Census Tract TIGER/Line*
Temperature *[°C]*	Temperature	2012–2024	*BEACO*_*2*_*N*
Pressure *[kPA]*	Pressure	2012–2024	*BEACO*_*2*_*N*
Relative Humidity *[%]*	Relative Humidity	2012–2024	*BEACO*_*2*_*N*

Land use data for the study area was downloaded from the global ESRI Sentinel-2 10 m Land Use/Land Cover database as a TIF image, where land use information is represented by pixels with unique integer values. Each integer value was then matched to its corresponding land use label. The California General Land Use Plan is another source of available land use data. Given limitations around spatial coverage, this data was only used to supplement information about industrial sites in the study region, represented as polygon geometries. Road and annual average daily traffic (AADT) data were downloaded as shapefiles, where roads are represented as lines and AADT information is stored in point geometries. NDVI data was calculated using Landsat 8–9 OLI Collection 2 Surface Reflectance TIF images, where pixels contain NDVI information as float values between − 1.0 and 1.0. Finally, population density data by census tract was represented by polygon geometries.

Geographic coordinates for the BEACO_2_N sensor locations were converted into point geometries and overlaid with spatial feature data. Buffers were created around each node to extract relevant feature data. The buffer radii used were 50, 100, 200, 300, 500, 1000, 2000, 3000, 4000, and 5000 m. Spatial feature data was extracted within each of these ten buffer zones for twelve selected features: built area, rangeland, trees, water, bare ground, crops, flooded vegetation, industrial areas, annual average daily traffic (AADT), road length, normalized difference vegetation index (NDVI), and population density. Feature extraction methods were primarily based on Lee et al. (2017) and are summarized in Table 1. This process yielded a total of 120 spatial features (12 variables across 10 buffer radii).

Temperature, pressure, and relative humidity data was collected alongside CO_2_ by BEACO_2_N sensors; these meteorological features have the same temporal resolution as the CO_2_ data and were aggregated as daily averages alongside CO_2_. By contrast, daily data for spatial features described above was not available, and the temporal resolution of these features is instead constant. While land use, NDVI, and population density features are relatively stable over time, the constant temporal resolution for traffic data is a greater compromise. Since the meteorological features lack spatial coverage beyond their point geometries, they were not processed using buffers. BEACO_2_N data for CO_2_ and meteorological conditions were merged with buffered feature data using node_id as a common feature, yielding the final dataset of 123 potential numerical feature variables and CO_2_ data as the target variable. All features were scaled to have a mean of 0 and standard deviation of 1.

### Feature selection

The feature selection approach was informed by relevant studies, including Larkin et al. (2023), Lee et al. (2017), Li et al. (2021), and Wong et al. (2021). In this study, Spearman’s correlation coefficient was used to identify key features, as it does not require a linear relationship between variables, making it more suitable for non-linear models. This coefficient measures the strength and direction of the relationship between predictors and target variables, with values ranging from − 1.0 to 1.0. Features with an absolute Spearman’s correlation coefficient above 0.03 were selected for modelling inputs. Additionally, a variable inflation factor (VIF) with a cutoff of 3 was applied to further reduce multicollinearity between features (Lee et al. 2017; Li et al. 2021; Wong et al. 2021). Features that contained all zero observations were excluded from the analysis.

### Model description

**Fig. 2:**
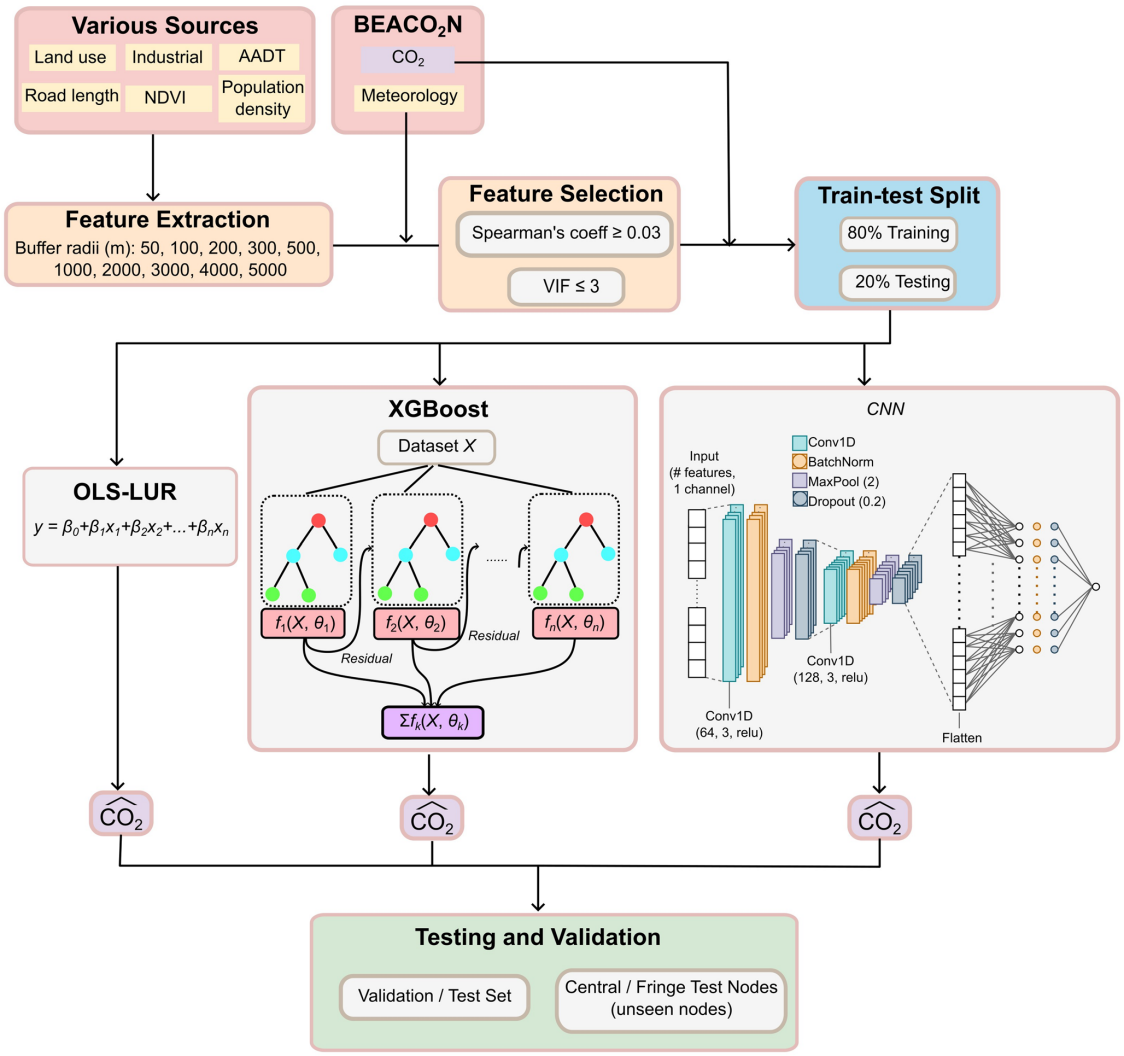
The modelling workflow. Data collection (red), data processing by feature extraction and feature selection (orange), train-test split (blue), modelling using traditional LUR, XGBoost, and CNN (grey) and model evaluation (green). XGBoost illustration is adapted from Guo et al. 2020

The modelling workflow is illustrated in Fig. 2. This section explores details around the three models (LUR, XGBoost and CNN) in greater depth. It is noted here that random forest (RF), a tree-based model similar to XGBoost, was explored as a modelling approach but excluded due to its lower efficiency and performance. In a preliminary test, an RF model with same number of trees performed worse than XGBoost and was therefore excluded from further modelling and evaluation. Instead, a CNN model was chosen in addition to the tree-based XGBoost model for its ability to capture spatial relationships across sensor data. The use of such a 1D CNN model on an unstructured grid of nodes has been investigated previously, for example, by Cheng et al. 2020.

#### Traditional LUR (Ordinary least squares)

Traditional LUR observed in literature consists of a multiple linear regression model used to explore the relationship between predictors (such as land use type, emission source, and meteorological conditions) and air pollution. In this study, the method is applied to predict CO_2_ concentration. Specifically, ordinary least squares (OLS) was used to fit a multiple linear regression model to the selected features and the target CO_2_ concentrations.

#### Extreme gradient boosting (XGBoost)

XGBoost is an ensemble decision tree model that leverages gradient boosting for improved computational speed and predictive performance. Wong et al. (2021) explored XGBoost in the context of LUR by “incorporating” the two methods for carbon monoxide prediction. In their combined approach, LUR was used for feature selection, by fitting multiple linear regression models between the feature and target variables, exploring correlation strength, and evaluating the significance and direction of feature coefficients. However, using LUR for feature selection imposes a linear relationship on the relationship between features and the target variable, thereby limiting XGBoost’s capacity to capture non-linear patterns. In this study, features were instead selected using the protocol described in Sect. 2.3, which is based on Spearman’s correlation coefficient and variance inflation factor (VIF). Gradient descent was used to minimize the loss function, defined as mean squared error (MSE). The XGBoost regression model was trained using grid search with cross-validation to optimize key hyperparameters. The final model configuration was selected based on cross-validated performance on the training set. The best-performing XGBoost model consisted of 300 trees with a maximum depth of 5 and a learning rate of 0.05.

#### Convolutional neural networks (CNN)

CNN has been used to capture complex relationships within input data for classification or regression tasks. Here, a one-dimensional convolutional neural network (1D CNN) was trained to predict CO_2_ concentrations (Fig. 2). The selected feature data was reshaped to explore the relationship between predictor variables. The model architecture includes two convolutional layers, the first applying 64 filters and the second applying 128 filters, each with a kernel size of 3 and a ReLU activation function. The convolutional layers are each followed by a batch normalization layer to stabilize training, a max pooling layer with a pool size of 2 to reduce spatial dimensions, and a dropout layer with dropout rate of 20% to minimize overfitting. The output is then flattened and fed to a dense layer of 128 nodes activated using ReLU, followed by a batch normalization and a 20% dropout layer. The final output layer is a single node representing predicted CO_2_ concentrations. The CNN was compiled with the Adam optimizer, an initial learning rate of 0.001 and an MSE loss function. Early stopping, learning rate reduction on plateau, and model checkpointing based on validation loss were included as callbacks. CNN was trained for up to 150 epochs with a batch size of 64. The above CNN architecture was chosen based on hyperparameter tuning using a 3-fold cross-validated grid search.

### Model validation and testing

Model performance was evaluated using the coefficient of determination (R^2^), the root mean squared error (RMSE), mean squared error (MSE) and the mean absolute error (MAE) (Fig. 2).

#### Training nodes

To overcome the limitations associated with the temporal and spatial inconsistency in the BEACO_2_N data, the dataset was filtered so that each day was represented by at least 27 nodes while every node reported at least 200 daily averages. This produced a final dataset 17,389 daily CO_2_ averages reported by 42 sensors. This data was randomly split into training, validation and test data: a total of 11,128 observations were used for training (64% of the data), 2,783 observations for validation (16% of data), and 3,478 observations were reserved for testing (20% of the data).

#### Unseen nodes

 To evaluate the transferability of the proposed models to unseen locations, 12 nodes are held out from the training nodes (red and brown nodes in Fig. 1). These unseen nodes were split into two groups: 5 central test nodes (sensors that are surrounded by training nodes), and 7 fringe test nodes (sensors located on the fringes of the training network).

## Results and discussion

### Importance of features

Among the 105 non-zero features, 32 were selected based on Spearman’s correlation coefficient criteria. Of these, 11 met the VIF condition. The final features are: temperature, pressure, relative humidity, trees area (50 m), total road length (1000 m), total road length (200 m), built area (2000 m), total AADT (3000 m), flooded vegetation area (1000 m), industrial area (5000 m), and average NDVI (50 m). The Spearman’s correlation coefficients indicate that meteorological features and the area of trees had the strongest absolute correlations with CO_2_, followed by population density, built area, NDVI, road length, and AADT. Details on feature selection scores can be found in Table S1.

While feature selection before model training helped identifying possible important features as listed above, feature importances for the trained models were additionally explored. Feature importance metrics for individual predictors can be found in Table S2 for LUR model (partial R^2^), and in Figure S1 for XGBoost model (gain feature importance). A SHAP analysis (Lundberg and Lee 2017) of the XGBoost model can be found in Figure S2. Overall, the analysis of feature importances highlights the significant contribution of meteorological features, especially temperature and pressure, in predicting ambient CO_2_ concentrations. The importance of these features is likely due to their granular temporal resolution, which aligns with the target variables. In contrast, land use features are temporally invariable, remaining constant for all observations at a given node and buffer radius. While road length and AADT features were selected, their relative importance was lower than expected, considering findings from previous LUR studies (Larkin et al. 2023; Li et al. 2021; Ryan and LeMasters 2007; Wong et al. 2021). The inclusion of tree coverage improved the accuracy of both LUR and XGBoost models, though its low SHAP score indicates that it was not influential across the entire study area, as it was not abundant. Instead, built area, the most prevalent land use type, along with industrial activities in the surrounding areas, more consistently shaped CO_2_ predictions across the region. 

### Model evaluation

**Fig. 3:**
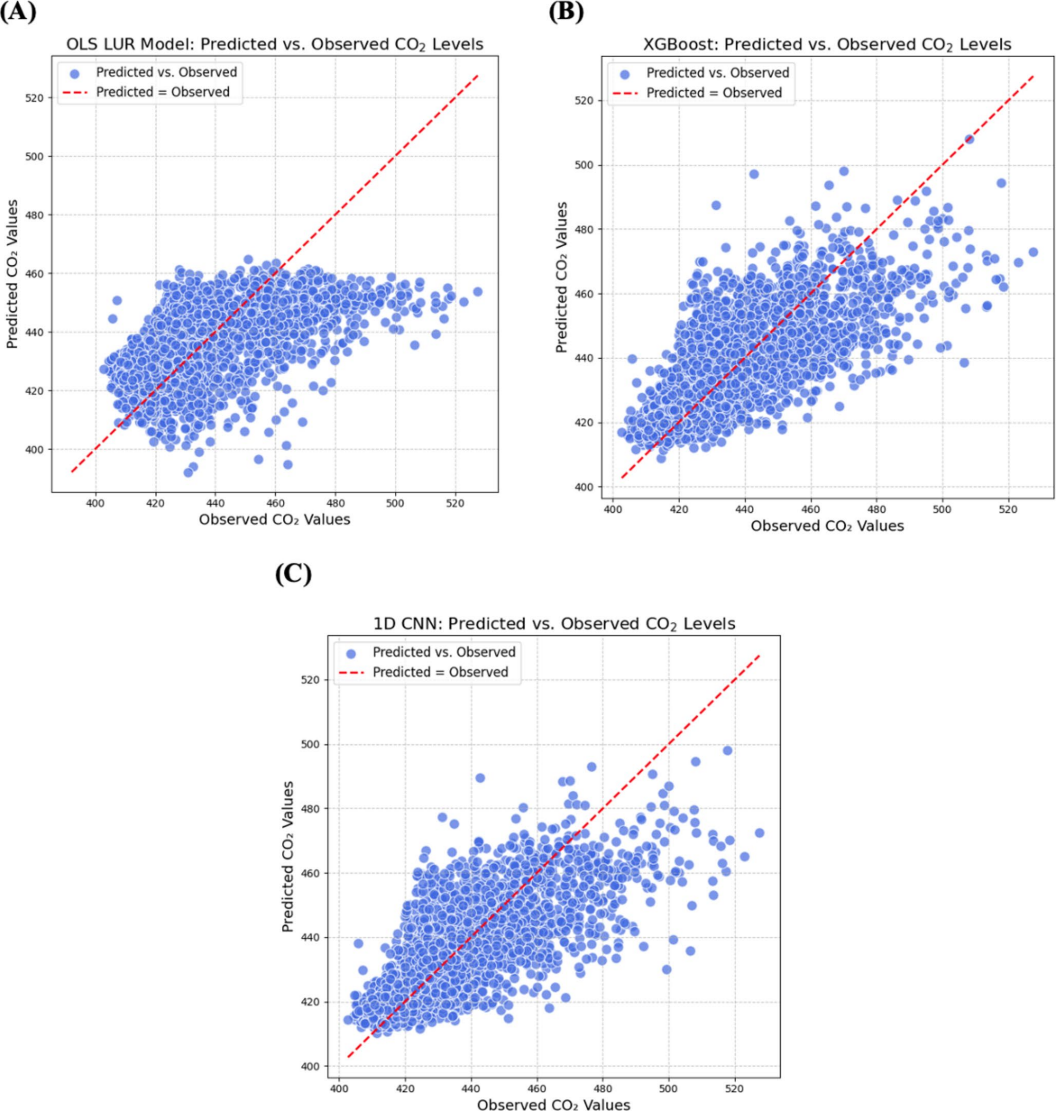
Model CO_2_ Predictions versus True Concentration Observations on training nodes. (**A**) LUR, R^2^ = 0.34. (**B**) XGBoost, R^2^ = 0.58. (**C**) CNN, R^2^ = 0.58

**Fig. 4:**
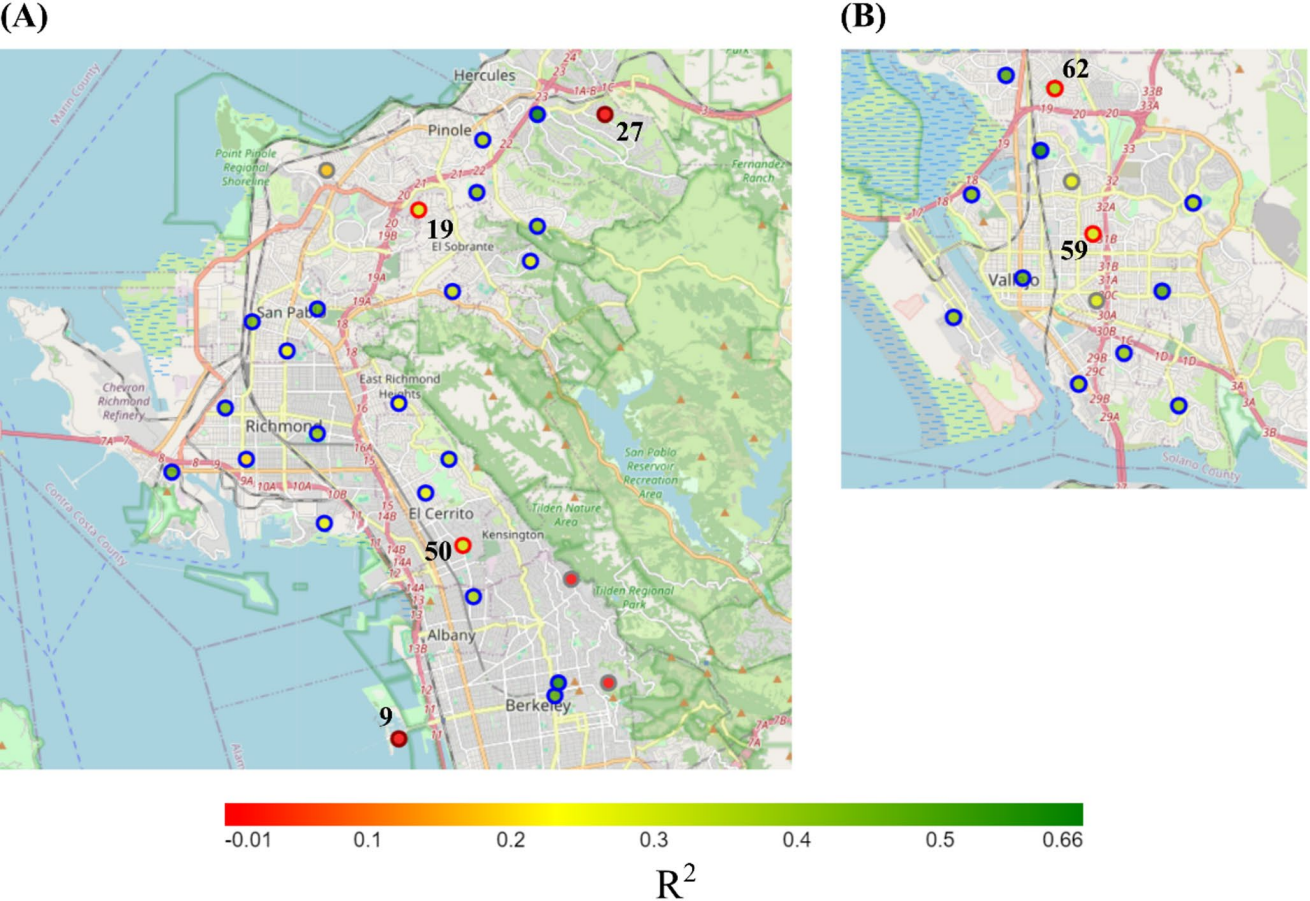
Correlation coefficients (R^2^) for individual nodes in two regions: (**A**) Richmond and north Berkeley; (**B**) Vallejo. Node_id for unseen nodes is marked near their positions: node_ids 9 and 27 are fringe test nodes; node_ids 19, 50, 59, and 62 are central test nodes. Results in the figure are for the CNN model. Colour of the node boundary indicates the node group: blue – training node; red – central test node; brown – fringe test node; grey – invalid node

The performance of the LUR and two ML-based models was evaluated for predicting ambient CO_2_ concentration in the San Francisco Bay Area. Table 2 summarizes the model evaluation scores using the reserved test data from training and unseen nodes while Fig. 3 shows performance comparison of individual observations. In Table 2, the results show that the linear LUR model explains 34% of the variability in the observed CO_2_ data, while both XGBoost and CNN account for 58%. The LUR model yielded RMSE, MSE, and MAE values of 15.81, 250.02, and 12.04, respectively. In comparison, XGBoost achieved RMSE, MSE, and MAE values of 12.56, 157.66, and 9.14, respectively, while CNN recorded RMSE, MSE, and MAE values of 12.63, 159.45, and 9.08. The R² values calculated at nodes are plotted in Fig. 4 for two regions: Richmond, North Berkeley, and Vallejo. Geographically, the R² values were highest for training nodes in the Richmond, North Berkeley, and Vallejo areas, where the sensor network was particularly dense and evenly distributed, compared to areas in San Francisco, Berkeley, and Oakland. For model performance on all individual nodes, see Figure S3.

**Table 2:** Model performance and comparison

Evaluation Step	Metric	LUR	XGBoost	CNN
Test cases on training nodes	R^2^ RMSE MSE MAE	0.34 15.81 250.02 12.04	0.58 12.56 157.66 9.14	0.58 12.63 159.45 9.08
Central Test Nodes	R^2^ RMSE MSE MAE	0.31 19.13 366.05 15.47	0.42 17.48 305.67 12.90	0.42 17.46 304.71 13.01
Fringe Test Nodes	R^2^ RMSE MSE MAE	−0.69 20.12 404.76 17.24	−0.88 21.24 451.14 18.11	−0.47 18.77 352.46 16.10

Overall, XGBoost and CNN outperformed the linear LUR model, indicating that non-linear models better capture the underlying relationships between environmental predictors and CO₂ concentrations (Fig. 3). Like the carbon monoxide models developed by Wong et al. (2021), this study demonstrates the superior performance of ML algorithms over traditional LUR, indicating that the non-linear trend is not limited to CO_2_ alone. This supports the argument that modelling intraurban ambient gases, such as air pollutants or greenhouse gases, should shift away from linear regression methods and instead leverage the potential of ML algorithms for more accurate predictions.

### Transferability on unseen nodes

Model evaluation on unseen nodes was conducted to assess the site-to-site transferability of the proposed models, with the results presented in Table 2. It can be observed that the models performed better for central test nodes, where XGBoost and CNN each explained 42% of the variability in the data, compared to the linear LUR model, which captured only 31% of the variability for these nodes. Overall, the CNN model achieved the best performance for unseen node locations across performance metrics, although its advantage over XGBoost was marginal. All three models performed poorly on the fringe test nodes (brown nodes in Fig. 1). The models therefore have a low external validity, which echoes conclusions reached about the transferability and generalizability of traditional LUR models (Larkin et al. 2023; Li et al. 2021). Figure 4 illustrates the performance of the CNN model on individual nodes. Comparing results between central and fringe test nodes shows that predictive accuracy is higher at unseen locations situated centrally within the training node network (e.g., node_ids 19, 50, 59, and 62) than at locations farther from the main cluster of training nodes (e.g., node_ids 9 and 27). This suggests that models predicting intraurban CO_2_ concentrations should be tailored to specific regions and provide fine spatial resolution coverage of the area. Poor performance at fringe nodes likely stems from their proximity to land use types that were underrepresented near training nodes, such as water bodies (node_id 9) and forested areas (node_id 27). This finding aligns with conclusions from Ryan and LeMasters (2007) regarding the significance of capturing land use variability in determining model performance.

### City layout impact and implications on urban planning

**Fig. 5:**
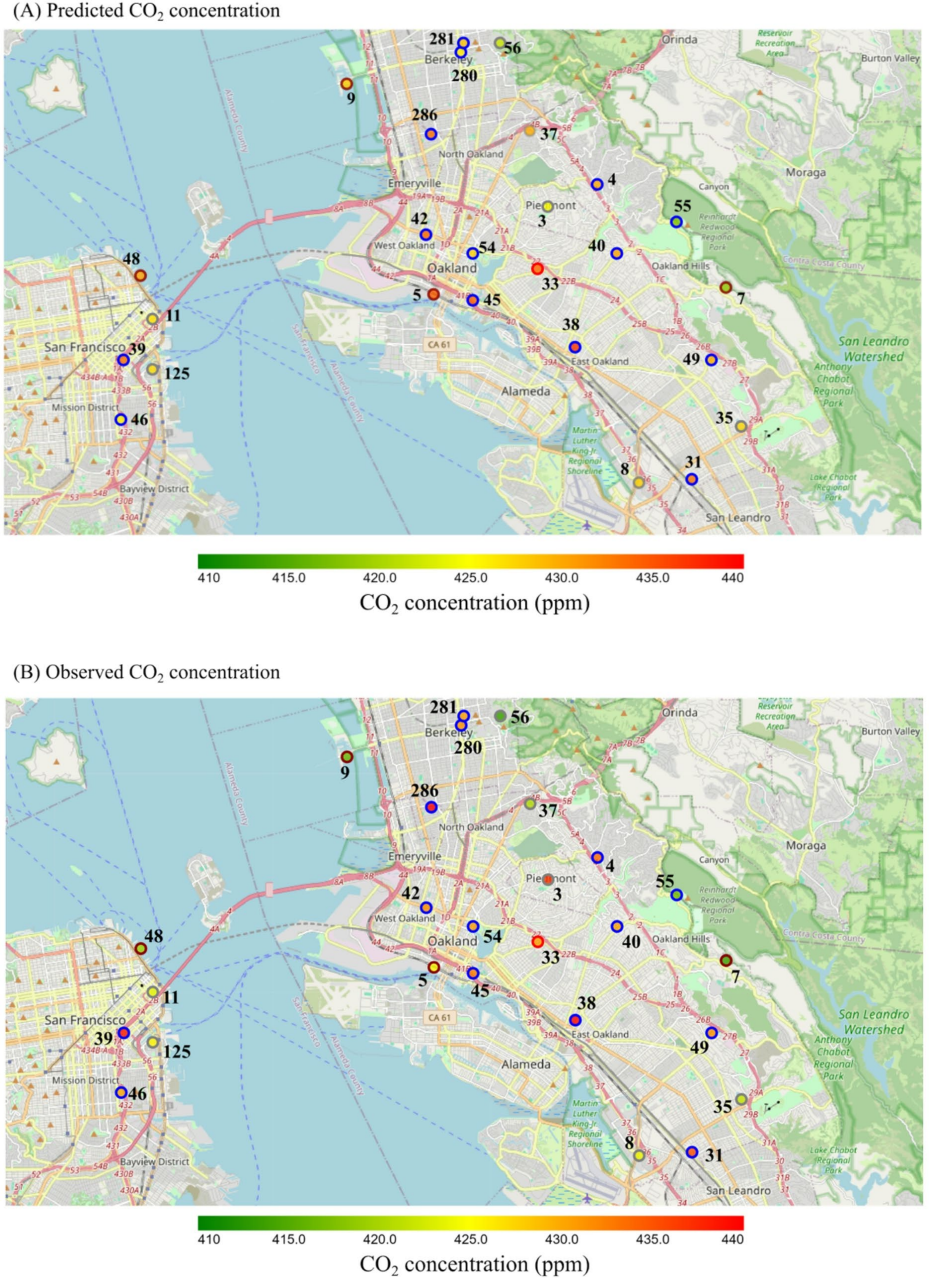
Predicted and observed CO_2_ concentration (ppm) around San Francisco-Oakland region. Averages of all daily concentrations are plotted. Nodes are coloured by the average concentration. Node IDs are marked near nodes. Predicted results are generated from the CNN model. Colour of the node boundary indicates the node group: blue – training node; red – central test node; brown – fringe test node; grey – invalid node

**Fig. 6:**
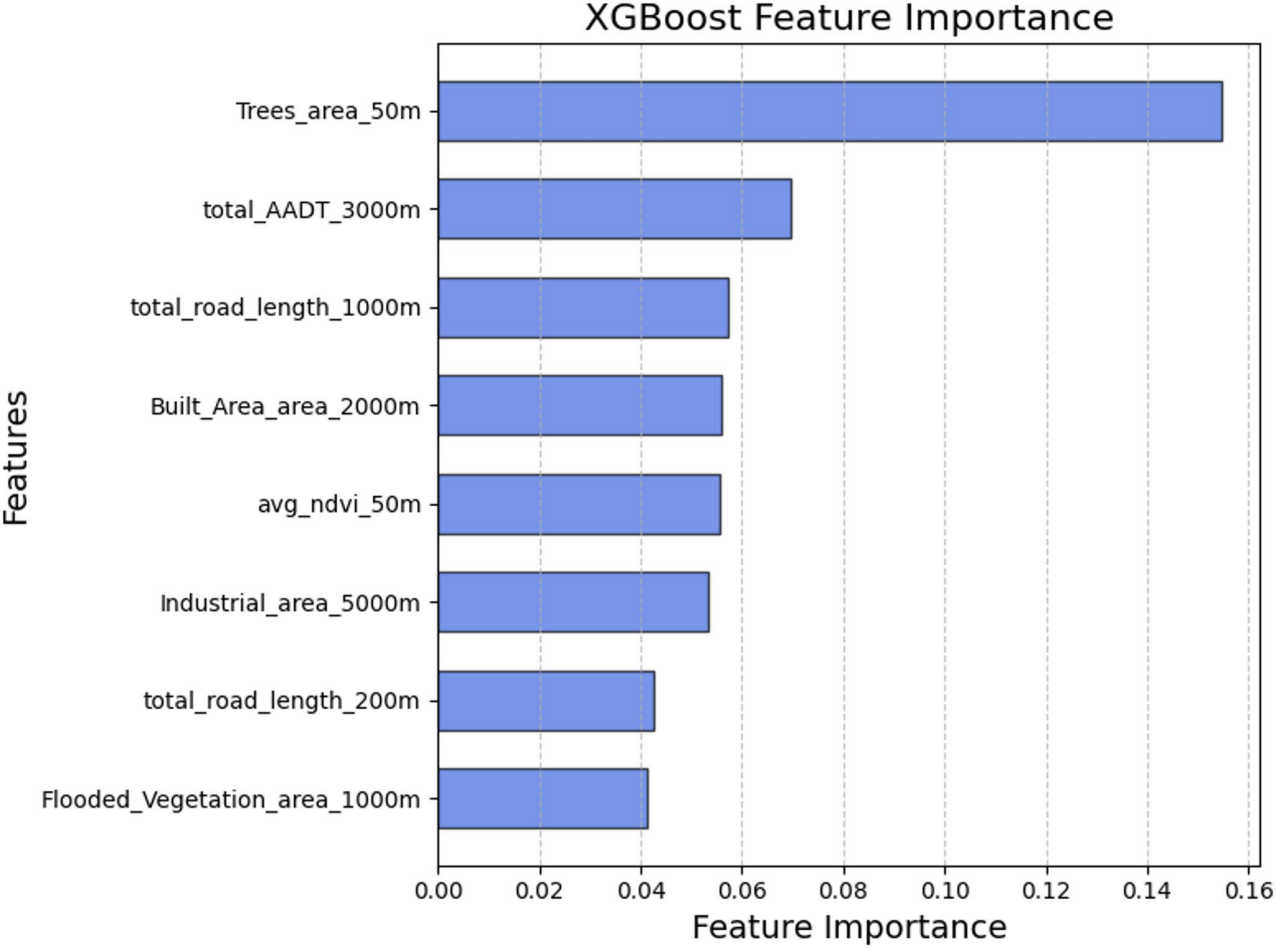
XGBoost Gain Feature Importance for spatial features related to city planning. The gain statistic describes the improvement in accuracy achieved by adding a given feature to the model

The predicted and measured daily average CO_2_ concentrations at sensor locations in San Francisco and Oakland is plotted in Fig. 5. Concentrations predicted by the CNN model aligned more closely with measured concentrations in areas with dense building coverage and near major roads, with CO_2_ concentrations exceeding 436 ppm (e.g., node_ids 31, 38, and 286). This suggests that emissions from traffic and residential/commercial buildings are the main contributors to elevated ambient CO_2_ levels. Conversely, sensors recording lower CO_2_ concentrations, below 416 ppm (e.g., node_ids 7, 37, 55, and 56), are situated near or within green areas, while others (e.g., node_ids 9 and 48) are located near water. This indicates that green and blue infrastructures play a significant role in supporting decarbonization efforts. It is noteworthy that the CNN model successfully captured the same trend across three nodes (node_ids 7, 55 and 56) in the green areas. The analysis of feature importance underscores the influence of city layouts on the spatial distribution of CO_2_. XGBoost modelling results (as shown in Fig. 6) identify trees within a 50 m buffer radius and total AADT within a 3000 m buffer radius as the two most critical spatial features (excluding meteorology) for predicting CO_2_ levels. These are followed by road lengths within a 1000 m buffer radius and built area within a 2000 m buffer radius.

Overall, these findings suggest that advancements in land use models can enhance urban CO_2_ prediction, identify CO_2_ emission sources, and assist planners in prioritizing areas for green and blue spaces while improving the built environment.

### Limitation of this study

The primary limitation of this study lies in the quality and availability of data for intraurban CO_2_ and predictor variables, highlighting the need for a more representative monitoring network to support urban planning. While the BEACO_2_N network offers superior spatiotemporal resolution compared to other intraurban CO_2_ monitoring networks, inconsistent data validity across nodes and time introduced significant spatiotemporal imbalances that had to be addressed. Additionally, the network’s generalizability was limited, particularly in areas with land use types underrepresented during the training process. The irregular and coarse spatial distribution of sensors also contributed to poorer model performance in certain areas, unlike Richmond and Vallejo, where the sensor network was denser and more uniform. Another key limitation was the fixed temporal resolution of environmental features, especially traffic data. Ideally, predictor variables should be collected with the same spatial and temporal resolution as CO_2_ data.

## Conclusions

This study was the first of its kind to evaluate the viability of using LUR to predict intraurban ambient CO_2_ concentrations. The study has demonstrated that ML-based algorithms can outperform traditional LUR in terms of predictive accuracy, illustrating that the relationship between environmental features and CO_2_ should not be presumed to be linear. This conclusion corroborates the findings of a previous study, underscoring the potential and value of expanding upon traditional LUR using novel modelling approaches. Evaluating models using data from unseen node locations further illustrated the models’ potential site-to-site transferability at the locations situated centrally within the training node network, but poor performance at fringe locations. The availability and spatiotemporal variability of data from intraurban CO_2_ monitoring networks is scarce and remains a major limitation for research in this field. In light of rising urbanization and the increasingly drastic effects of climate change, efforts to establish intraurban CO_2_ monitoring networks with high spatiotemporal resolution should be expanded. The network should monitor both environmental variables and predictor variables such as traffic data at the same time. More research into the modelling of ambient intraurban CO_2_ is necessary to better understand and predict the effects of anthropogenic urban activities and land use change on climate change. This research is vital to inform decision-makers on sustainable development and urban decarbonization in the 21 st century.

## Supplementary Information

Below is the link to the electronic supplementary material.

Supplementary Material 1 (PDF 315 KB)

## Data Availability

No datasets were generated or analysed during the current study.
