# Supplementary material for: Machine learning-based land-use regression models for predicting carbon dioxide concentrations in San Francisco Bay area
Source: Environ Earth Sci. 2025 Sep 26;84(19):539. doi: 10.1007/s12665-025-12582-w (PMC12474649; doi:10.1007/s12665-025-12582-w)
Supplement: Supplementary file 1 — Supplementary Material 1 (PDF 315 KB) [file 12665_2025_12582_MOESM1_ESM.pdf]

Machine learning-based land-use regression models for predicting carbon dioxide concentrations in San Francisco Bay Area

Anna C Smith[a], Linfeng Li[a, *], Jiansheng Xiang[a], Fangxin Fang[a]

[a] *Department of Earth Science and Engineering, Imperial College London, SW7 2AZ, London, UK*

* *Corresponding author: l.li20@imperial.ac.uk (L. Li).*

Supplementary Material

Table S1 Feature selection scores and outcomes. 32 features selected by Spearman's correlation coefficient, of which 11 were selected using VIF.

Feature	Spearman	VIF
temp	-0.51	1.26
pressure	0.40	2.47
rh	-0.11	1.20
Trees_area_100m	-0.09	-
Trees_area_50m	-0.08	2.14
Trees_area_200m	-0.06	-
Trees_area_300m	-0.06	-
Trees_area_500m	-0.05	-
avg_pop_dens_2000m	0.05	-
avg_ndvi_100m	-0.05	-
Built_Area_area_1000m	0.05	-
avg_pop_dens_3000m	0.05	-
avg_pop_dens_4000m	0.04	-
Built_Area_area_3000m	0.04	-
Built_Area_area_4000m	0.04	-
avg_ndvi_200m	-0.04	-
avg_pop_dens_1000m	0.04	-
total_road_length_1000m	0.04	1.65
Trees_area_1000m	-0.04	-
avg_ndvi_300m	-0.04	-
total_road_length_200m	0.04	1.46
Built_Area_area_2000m	0.04	1.93
avg_ndvi_500m	-0.04	-
avg_pop_dens_5000m	0.04	
total_AADT_3000m	0.04	1.40
Flooded_Vegetation_area_1000m	-0.03	1.22
Industrial_area_5000m	0.03	1.53
Built_Area_area_500m	0.03	-
total_AADT_1000m	0.03	-
avg_pop_dens_500m	0.03	-
avg_ndvi_50m	-0.03	1.37
avg_ndvi_1000m	-0.03	-

Table S2 Traditional LUR model feature statistics to help demonstrate feature importance

Feature	Coefficient	Standard Error	p-value	VIF	Partial R2
temp	-7.72	0.15	<0.005	1.25	0.16
pressure	10.00	0.21	<0.005	2.52	0.14
rh	-2.98	0.15	<0.005	1.20	0.03
Trees_area_50m	5.46	0.20	<0.005	2.17	0.05
total_road_length_1000m	-0.78	0.17	<0.005	1.66	<0.005
total_road_length_200m	0.34	0.16	0.04	1.47	<0.005
Built_Area_area_2000m	1.87	0.19	<0.005	1.93	0.01
total_AADT_3000m	0.34	0.16	0.03	1.40	<0.005
Flooded_Vegetation_area_1000m	-0.62	0.15	<0.005	1.22	<0.005
Industrial_area_5000m	-1.91	0.17	<0.005	1.54	0.01
avg_ndvi_50m	0.31	0.16	0.05	1.39	<0.005

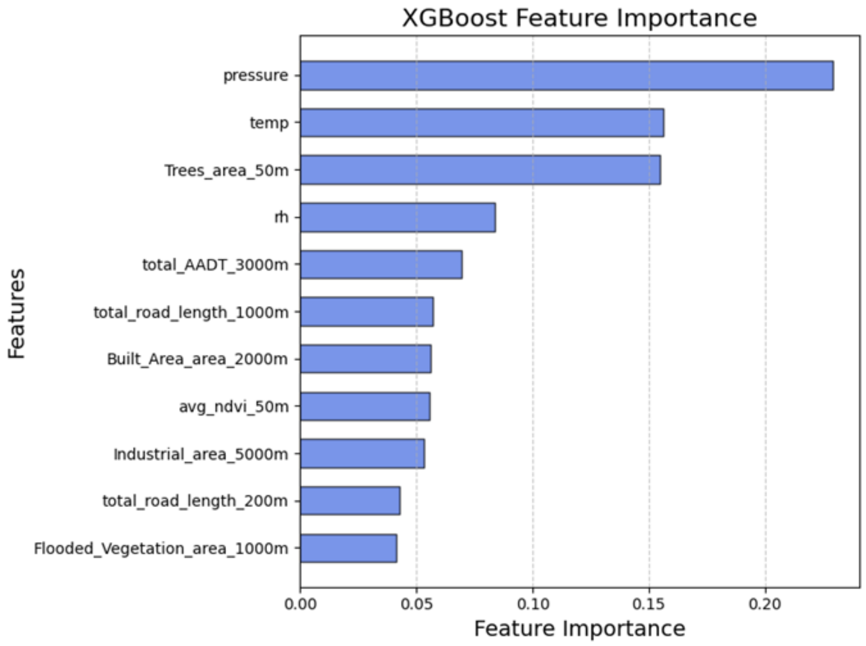

Figure S1 XGBoost Gain Feature Importance. The gain statistic describes the improvement in accuracy achieved by adding a feature to the model.

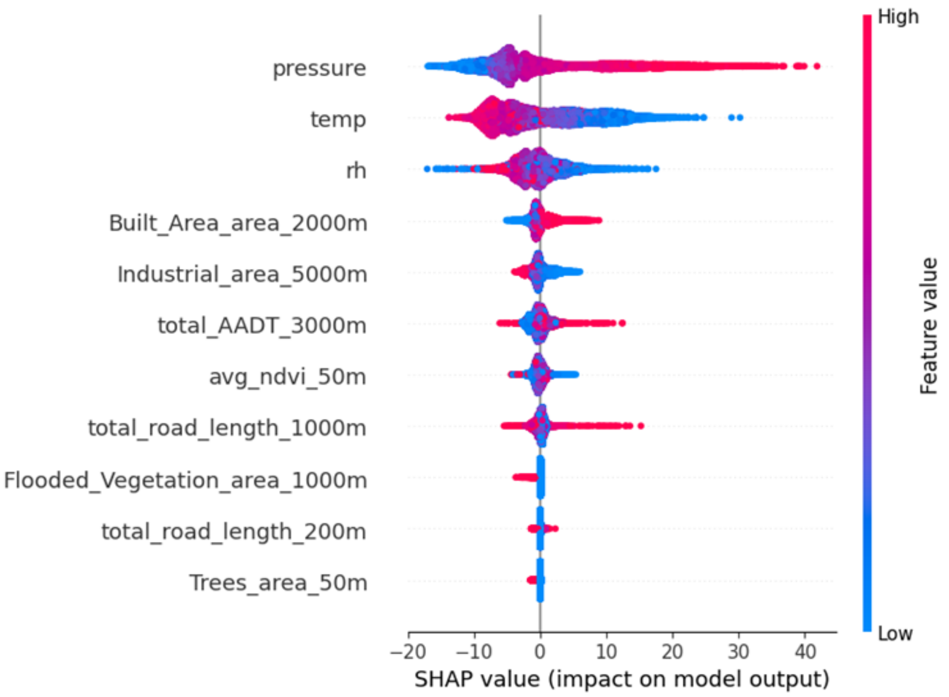

Figure S2 XGBoost SHAP Feature Importance. The SHAP values describe the contribution of each feature to shaping the model's predictions.

Figure S3 Interactive maps of model performance for individual nodes (https://ese-msc-2023.github.io/irp-acs223/results_explorer.html). (a) LUR model; (b) XGBoost model; (c) CNN model.
